# The effects of the COVID‐19 pandemic on French‐Canadian adolescents' academic motivation: A follow‐up study

**DOI:** 10.1002/ccr3.6376

**Published:** 2022-09-22

**Authors:** Jonathan Smith, Fanny‐Alexandra Guimond, Jérôme St‐Amand, Caroline Fitzpatrick, Julie Bergeron, Mathieu Gagnon

**Affiliations:** ^1^ Department of Preschool and Primary Education University of Sherbrooke Sherbrooke Quebec Canada; ^2^ School of Psychology University of Ottawa Ottawa Ontario Canada; ^3^ Department of Educational Sciences University of Quebec at Outaouais Gatineau Quebec Canada

**Keywords:** academic motivation, adolescents, COVID‐19 pandemic, expectancy‐value, high school

## Abstract

We previously shared results suggesting that the academic motivation of a sample of French‐Canadian adolescents remained stable from few weeks before the first wave to the second wave of the COVID‐19 pandemic. We here examine if this pattern persisted using data collected at a third time point.

## 1 INTRODUCTION

In an article published in early 2021,^1^ we used an expectancy‐value theoretical framework^2^ to assess the effects of the first and the second waves of the COVID‐19 pandemic on the academic motivation of a small sample of 90 French‐Canadian adolescents. More precisely, changes in their expectancy (i.e., competence beliefs) and value (i.e., interest in learning, utility value of learning, mastery goal orientation, and performance goal orientation) perceptions have been investigated based on their answers to a questionnaire. The questionnaire was administered at two time points, namely few weeks before the first positive cases of COVID‐19 infection were detected in the province of Quebec, Canada, and during the second wave of the pandemic. Findings revealed that most of the dimensions of their motivation remained somewhat stable during this period. In fact, only one undergone a (positive) change, namely their interest in learning. These results, which do not coincide with the usual declining pattern,^3^, ^4^, ^5^, ^6^ suggested that, when adolescents were allowed to reintegrate school after a prolonged home confinement, they were potentially relieved and even satisfied of being given the opportunity to interact in person again. As a result, they might have shown more enthusiasm toward school activities than usual. However, these results only represented a snapshot of the effects of the COVID‐19 pandemic and it was worth wondering if they were not underestimating long‐term potentially damaging effects. To have a better portray of the effects of this health crisis on adolescents' academic motivation, the participants were surveyed once more. Therefore, we here present the results of the analysis of this additional data.

## 2 METHOD

### 2.1 Design and sample

Most participants who previously answered the questionnaires at the two first time points agreed to answer it again. Indeed, of the initial sample of 90 adolescents, 78 participated at the third time point (attrition = 13.3%). These 78 students in 9th and 10th grade attended two urban public high school in the Greater Region of Quebec City (Quebec, Canada).

The first data collection took place in January 2020, few weeks prior to the initial outbreak of the COVID‐19 pandemic in Canada and the adoption of health regulations and emergency protocols. The second data collection was performed in November 2020, while a second wave of infections was underway. Adolescents were then reintegrating their school after experiencing distance learning activities for the entire months of September and October. The third and final data collection was conducted in May 2021, during a third wave of infections, while they keep benefitting from in class activities.

The same questionnaire used at the two first time points was completed a third time and allowed to document adolescents' expectancies of success (i.e., competence beliefs) and the value they place on learning (i.e., interest in learning, utility value of learning, mastery goal orientation, and performance goal orientation). They were presented statements related to these dimensions, and they had to indicate their level of agreement by selecting an answer corresponding to their perception.

### 2.2 Measures

#### 2.2.1 Competence beliefs

Competence beliefs were measured using four items adapted from the work of Ntamakiliro et al.^7^: “I'm proud of my grades at school,” “I'm as good as others at school,” “I'm not very good at school,” and “I'm not as good as others at school.” Items were rated on a six‐point scale ranging from 1 (*Totally disagree*) to 6 (*Totally agree*). The last two items were reverse coded, and all item scores were averaged to create a global score of student's competence beliefs (*α* = 0.85).

#### 2.2.2 Interest in learning

Interest in learning was measured using four items adapted from the work of Ntamakiliro et al.^7^: “I'm interested in what we learn at school,” “I like going to school,” “What we do in class is interesting,” and “I'm often bored in class.” Items were rated on a six‐point scale ranging from 1 (*Totally disagree*) to 6 (*Totally agree*). The last item needed to be reverse coded, and all item scores were averaged to create a global score of interest in learning (*α* = 0.82).

#### 2.2.3 Utility value of learning

Utility value of learning was measured using four items adapted from the work of Ntamakiliro et al.^7^: “What we learn in school will be useful in life,” “What we learn in school will be helpful in the future,” “What we learn in school is useful,” and “What we learn in school is not useful.” Items were rated on a six‐point scale ranging from 1 (*Totally disagree*) to 6 (*Totally agree*). The last item needed to be reverse coded, and all item scores were averaged to create a global score of utility value of learning (*α* = 0.87).

#### 2.2.4 Mastery goal orientation

The mastery goal orientation was measured using three items adapted from the work of Bouffard et al.^8^: “It's important for me to understand what we're learning at school,” “Understanding as much as possible is the most important thing for me at school,” and “I want to learn as much as possible at school.” Items were rated on a six‐point scale ranging from 1 (*Totally disagree*) to 6 (*Totally agree*). All item scores were averaged to create a global score of mastery goal orientation (*α* = 0.81).

#### 2.2.5 Performance goal orientation

The performance goal orientation was measured using four items adapted from the work of Bouffard et al.^8^: “My main goal in school is to be the best,” “It's important to me to be one of the best in my class,” “My main goal in school is to get good grades,” and “It's important to me to be better than other students.” Items were rated on a six‐point scale ranging from 1 (*Totally disagree*) to 6 (*Totally agree*). Item scores were averaged to create a global score of performance goal orientation (*α* = 0.86).

### 2.3 Plan of analysis

After performing a multiple imputation method with 25 iterations to address item‐level missing data, a repeated measure multivariate analysis of variance (MANOVA) with time as the repeated measure was conducted.

## 3 RESULTS

As can be seen in Table 1, the MANOVAs revealed a significant effect of time *F*(10.80) = 4.04, *p* < 0.001. Indeed, interest in learning increased from Time 1 (*M* = 3.78, *SD* = 0.85) to Time 2 (*M* = 4.01, *SD* = 0.85), but then decreased at Time 3 (*M* = 3.67, *SD* = 0.95). A similar pattern of results emerges for utility value which decreased from Time 2 (*M* = 4.40, *SD* = 0.82) to Time 3 (*M* = 4.02, *SD* = 0.92) as well as for mastery goal orientation which also decreased from Time 2 (*M* = 4.89, *SD* = 0.81) to Time 3 (*M* = 4.65, *SD* = 0.75). In reference to Cohen’s^9^ indications, this extra measure therefore allows to discern a large time effect on interest in learning and utility value. As for the time effect on mastery goal, it would be of moderate size.

**TABLE 1:** Results of repeated‐measure multivariate analyses of variance (MANOVAs).

Variable	*M* (SD)			Time	*F* (Effect size)
	T1	T2	T3		
Competence beliefs	4.61 (0.94)	4.66 (0.92)	4.52 (0.84)	1–2	0.33 (0.00)
				1–3	0.62 (0.01)
				2–3	2.00 (0.02)
Interest in learning	3.78 (0.85)	4.01 (0.85)	3.67 (0.95)	1–2	8.74** (0.09)
				1–3	1.45 (0.02)
				2–3	18.42** (0.17)
Utility value of learning	4.30 (0.88)	4.40 (0.82)	4.02 (0.92)	1–2	1.41 (0.02)
				1–3	9.91** (0.10)
				2–3	19.19** (0.18)
Mastery goal orientation	4.76 (0.86)	4.89 (0.81)	4.65 (0.75)	1–2	2.39 (0.03)
				1–3	1.39 (0.02)
				2–3	9.76** (0.10)
Performance goal orientation	3.43 (1.27)	3.35 (1.20)	3.48 (1.17)	1–2	0.84 (0.01)
				1–3	0.18 (0.00)
				2–3	2.25 (0.03)

*Note*: *n* = 78.

**p < 0.01.

## 4 DISCUSSION

The results offer new insight on the consequences of the COVID‐19 pandemic on adolescents' motivation. Indeed, they highlight potential negative changes that were not detected in our previous study where adolescents were possibly reacting positively to their return to in‐presence activities after home confinement. Indeed, in that study, competence beliefs, utility value of learning, mastery goals, and performance goals indeed remained somewhat intact, while interest in learning even increased.^1^ However, these new findings suggest that the previously found patterns were temporary. Indeed, in the longer run (by the end of the school year), not only adolescents' interest in learning decreased, but so did their utility value and mastery goals. These results are consistent with previous studies^10^, ^11^ which investigated fluctuations in motivation toward schoolwork during the COVID‐19 pandemic and particularly while adolescents experienced an alternation between remote teaching and in‐class activities. Usher et al.^11^ suggested that youth may have revised their priorities and, instead of focusing on learning opportunities, they sought to preserve their health and safety as well as the one of relatives they care for.

That being said, and as highlighted by Klootwijk et al.,^10^ it is difficult to determine whether such motivational changes are due to the COVID‐19 pandemic, especially since students' motivation tends to decline within a school year and across grade levels.^1^, ^2^, ^3^, ^4^ However, as we already pointed out, several recent studies revealed that adolescents' mental health has been negatively impacted by the COVID‐19 pandemic^12^, ^13^, ^14^, ^15^, ^16^, ^17^, ^18^ and our findings leave to think that their motivational resources were also impacted.

## 5 CONCLUSION

In sum, the results of this follow‐up study seem to be less situational and may reflect potential chronic effects of the COVID‐19 pandemic on adolescents' academic motivation. The findings are in line with several recent studies indicating that youth experienced a decrease in motivation toward schoolwork^10^, ^11^ as well as high levels of distress during the COVID‐19 pandemic.^12^, ^13^, ^14^, ^15^, ^16^, ^17^, ^18^ Consequently, it is likely that they had difficulties engaging in activities that, in normal circumstances, were salient to them. It remains to be seen if these negative effects on adolescents' motivational resources will persist in time.

## AUTHOR CONTRIBUTIONS

JS is the corresponding author. He managed the study, and he wrote and edited the manuscript. FAG assisted with the writing and editing of the manuscript. JSA, CF, and JB assisted with the editing of the manuscript. MG was involved in the management of the study.

## CONFLICT OF INTEREST

We have no conflicts of interest to declare.

## ETHICAL APPROVAL

The research received ethical approval from University of Sherbrooke.

## CONSENT

Written informed consent was obtained from participants (and their parents) to publish this report in accordance with the journal's patient consent policy.

## Data Availability

Data that support the finding of this study are available upon request.
